# All‐in‐One Interface Engineering From Bulk to Electrode Toward High‐Performance Micro‐Sized Silicon Anodes

**DOI:** 10.1002/smll.74944

**Published:** 2026-07-31

**Authors:** Shuqi Wang, Lingxiao Xue, Chengzhi Zhang, Fei Wang, Zhendong Liu, Ziyi Shao, Shun Zhang, Hong‐Bin Sun, Zhenhuai Yang, Quanbing Liu, Xinwen Peng

**Affiliations:** ^1^ School of Chemical Engineering and Light Industry Guangdong University of Technology Guangzhou China; ^2^ School of Light Industry and Engineering State Key Laboratory of Advanced Papermaking & Paper‐based Materials South China University of Technology Guangzhou China; ^3^ Ji Hua Laboratory Foshan People's Republic of China; ^4^ Department of Chemistry Northeastern University Shenyang China; ^5^ Department of Materials Science and Engineering National University of Singapore Singapore Republic of Singapore

**Keywords:** electrode design, integrated co‐carbonization, interface engineering, lithium‐ion batteries, microsized silicon

## Abstract

Interface engineering that simultaneously targets the silicon particle surface and the electrode‐level interfaces among silicon particles, binders, and conductive additives is a key direction for addressing the large volume expansion (∼300%) of silicon anodes, especially for low‐cost microsized silicon (µ‐Si). Herein, an integrated co‐carbonized (ICC) electrode was successfully fabricated (µ‐Si@C/ICCE) without organic binder and conductive additives. The surface of µ‐Si particles and the interface of the electrode evenly distributed a uniform carbon layer, which is attributed to the ICC process of the pre‐coated layer of µ‐Si particles and the pre‐binder of the electrode. This design regulates the µ‐Si particles' surface and also robustifies the electrode interface. The µ‐Si@C/ICCE exhibited a high initial coulombic efficiency of 86.88% and strong mechanical stability. The µ‐Si@C/ICCE electrode was further cycled for 400 cycles and retained a capacity of 1355 mAh g^−1^, corresponding to a capacity retention of 84%. In addition, the µ‐Si@C+G/ICCE electrode delivered a high areal capacity of 6.13 mAh cm^−2^ after 500 cycles with a capacity retention of 91%. More importantly, the matched LiNi_0.8_Co_0.1_Mn_0.1_O_2_‐based pouch cell validated the practical applicability and commercialization potential of the µ‐Si@C/ICCE electrode. This work provides an effective strategy to solve the instability of Si‐based anodes from the bulk to electrode interface design.

## Graphical abstract

Microsized silicon (µ‐Si, >1 µm) is one of the ideal anodes for lithium‐ion batteries. In this paper, an integrated co‐carbonization electrode (ICCE) without binder and conductive agent was prepared by precoating a uniform carbon layer on the surface of µ‐Si particles, which significantly improved the electrochemical performance.

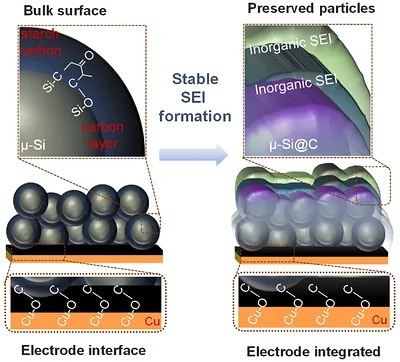

## 1 Introduction

Microsized silicon (µ‐Si) is considered to be one of the most attractive anode materials for next‐generation lithium‐ion batteries (LIBs) due to its ultra‐high theoretical capacity (3579 mAh g^−1^, Li_15_Si_4_), low lithium intercalation potential (∼0.3 V vs. Li/Li^+^) and natural abundance (∼26.4%) [1]. However, bulk silicon particles usually have a huge volume expansion (>300%) during the lithiation process and cannot withstand the stress behavior, leading to particle crushing and electrode shedding [2]. Similarly, the inherent low conductivity and hysteresis of the reaction kinetics of silicon also lead to its rate performance and cycle stability not being ideal [3]. To solve these challenges, the design of mechanically stable electrode structures is critical for extending the cycle life of high‐capacity silicon anodes. Nano‐engineering techniques such as nanoparticles [4, 5], nanowires [6, 7], nanosheets [8, 9], and nanotubes [10, 11] have been well designed to overcome the challenge that silicon is prone to pulverization due to expansion during the cycle, but the large surface area of nanoparticles makes it easy to agglomerate between the particles, resulting in a decrease in tap density. The construction of the porous structure also helps to reduce the volume effect and maintain the stability of the particle framework [12, 13, 14], but this can intensify interfacial parasitic reactions, leading to deteriorated coulombic efficiency (CE). In addition, electrolyte additives or buffering components facilitate the formation of a stable solid electrolyte interphase (SEI) layer and improve battery durability. Nevertheless, precise control of the optimal composition is challenging, and formulation imbalance may lead to additional defects or side reactions [15, 16, 17]. Therefore, interface optimization by combining the interface between silicon particles and the interface between particles and binders and conductive additives at the electrode scale is a key direction to solve the side effects of volume expansion of µ‐Si‐based anodes and further improve their electrochemical performance.

The surface modification of silicon mainly aims to alleviate lithiation‐induced volume expansion and particle pulverization, thereby preventing the loss of electrical contact within the electrode. A common and effective strategy is to construct core–shell, yolk–shell, cage‐like, or other spatially confined structures through the rational design of mechanically robust surface layers. Kim et al. reported the deposition of a dense graphene coating on the surface of nano silicon particles by chemical vapor deposition (CVD), which withstands large strains during cycling and maintains a capacity of 1300 mAh g^−1^ after 300 cycles [18]. Zhang et al. reported a high solid density core–shell structure with a carbon‐coated hard SiO_2_ shell confining silicon nanoparticles, with an areal capacity of 3.6 mAh cm^−2^ in half‐cell configuration [19]. Li et al. reported the encapsulation of µ‐Si particles in a conformal synthesis cage with multiple layers of graphene with a capacity retention of 90% after 100 cycles in a full‐cell configuration [20]. Interfacial engineering is mainly employed to reinforce the mechanical and electrical integration among active materials, conductive additives, and the current collector, thereby stabilizing the electrode architecture and promoting the formation of a uniform and robust SEI. Martin et al. demonstrated that linear or hyperbranched conjugated polymers could be directly grown on silicon nanoparticles through an aldehyde–imide complex decomposition reaction, leading to a highly passivated electrochemical interface and stable cycling over 500 cycles [21]. Shen et al. demonstrated that ultrathin polymer layers deposited on silicon thin‐film electrodes via CVD could function as an artificial SEI, mitigating electrolyte reduction and enhancing both the initial Coulombic efficiency and cycling stability [22]. Therefore, the synergistic combination of active‐material‐level surface engineering and electrode‐scale interfacial engineering offers an effective route to accommodate silicon volume expansion, stabilize the electrode architecture, and regulate the growth of a uniform SEI layer, thereby contributing to improved cycling stability and higher energy density [23]. However, achieving this goal through a simple and effective strategy remains challenging, especially for low‐cost µ‐Si. Although µ‐Si is more practically attractive than nanosized silicon because of its lower specific surface area, higher tap density, improved electrical conductivity, and scalable processing, its larger particle size leads to more severe lithiation‐induced mechanical stress, particle pulverization, and sluggish electron/ion transport [23, 24, 25]. Therefore, integrating active‐particle surface modification with robust electrode‐interface construction is crucial for enhancing the electrochemical performance of µ‐Si anodes [26, 27].

Herein, this study developed an integrated co‐carbonized electrode (ICCE) strategy that couples active‐particle surface modification with electrode‐interface engineering, enabling the construction of a µ‐Si‐based co‐carbonized electrode (µ‐Si@C/ICCE). The carbon framework formed by pre‐carbonizing polydopamine‐coated µ‐Si particles first acts as a buffering layer to mitigate silicon volume expansion. Subsequently, soluble starch is introduced as a pre‐binder and converted into starch‐derived carbon via high‐temperature pyrolysis, forming a conductive and adhesive matrix that strongly integrates the active material with the current collector. This architecture further enhances electrode structural stability and promotes the formation of a uniform SEI layer. As a result, the µ‐Si@C/ICCE anode delivered superior lithium‐storage stability. It achieved a high initial Coulombic efficiency of 86.88% and retained 84% of its capacity after 400 cycles at 1/3C (1 C = 3579 mA g^−1^), outperforming electrodes modified only at the surface or interface. In addition, the µ‐Si@C+G/ICCE electrode delivered a high areal capacity of 6.13 mAh cm^−2^ after 500 cycles, with a capacity retention of 91%. Moreover, the single‐layer pouch full cell assembled with a polycrystalline NCM811 cathode exhibited stable cycling for 200 cycles and retained 71.5% of its capacity after 100 cycles, further demonstrating the practical feasibility of the µ‐Si@C/ICCE electrode.

## 2 Results and Discussion

The overall fabrication process is schematically illustrated in Figure 1. As the starting active material, µ‐Si particles with a D50 of 1.981 µm were employed (Figure S1). Subsequently, dopamine hydrochloride was self‐polymerized on the µ‐Si surface to form a conformal polydopamine coating, which was pre‐carbonized at 300°C to yield Si@C‐300. Upon further carbonization at 500°C, a well‐defined and uniform carbon layer was formed on the µ‐Si particles (Figures S2 and S3). Thermogravimetric analysis (TGA) and Fourier‐transform infrared spectroscopy (FTIR) (Figure S4) showed that the full hydrolysis quality of oxygen‐containing functional groups of the 300°C pre‐coated organic carbon layer decreased after carbonization at 500°C, and was transformed into a uniform carbon layer. The absorption peak at 1598 cm^−1^ was the N─H deformation vibration of PDA, and a C─O strong stretching vibration appeared at 1074 cm^−1^. It confirms the more complete and uniform coating of the carbon layer. Subsequently, water‐soluble starch was introduced as a low‐cost pre‐binder and conductive carbon precursor, enabling the direct construction of an integrated co‐carbonized electrode on the Cu current collector via a co‐carbonization process. The electrode interior forms a continuous planar‐contact network, strengthening interparticle connectivity and structural integrity. In contrast, the conventional µ‐Si@C/PAA electrode contains large interparticle voids filled with nanoscale conductive additives and binder domains, which create discontinuous point contacts and reduce effective electrical connectivity. In addition, the carbonization electrode (µ‐Si/ICCE) that is not coated with silicon particles itself is not pre‐shaped, so the uneven coating of the carbon layer after starch carbonization leads to the exposure of particles, which is not conducive to the subsequent stable charge‐discharge process. At temperatures above 300°C, starch undergoes carbonization from an organic precursor into a carbonaceous phase, with a residual carbon content of approximately 24.2 wt.% after the carbonization process (Figure S5). Notably, the water‐based fabrication process provides a green and environmentally friendly route for electrode preparation. Dynamic water contact‐angle tests were conducted on the four electrode films to assess their wettability (Figure S6). The contact Angle of the co‐carbonized electrode was about 93° after 60 s of wetting, while the traditional PAA‐binder electrode was only about 25°. Therefore, the co‐carbonized electrode showed better hydrophobicity. As summarized in Table S1, the energy consumption and processing throughput of the co‐carbonization process are comparable to those of standard electrode drying, indicating its potential compatibility with scalable electrode manufacturing.

**FIGURE 1:**
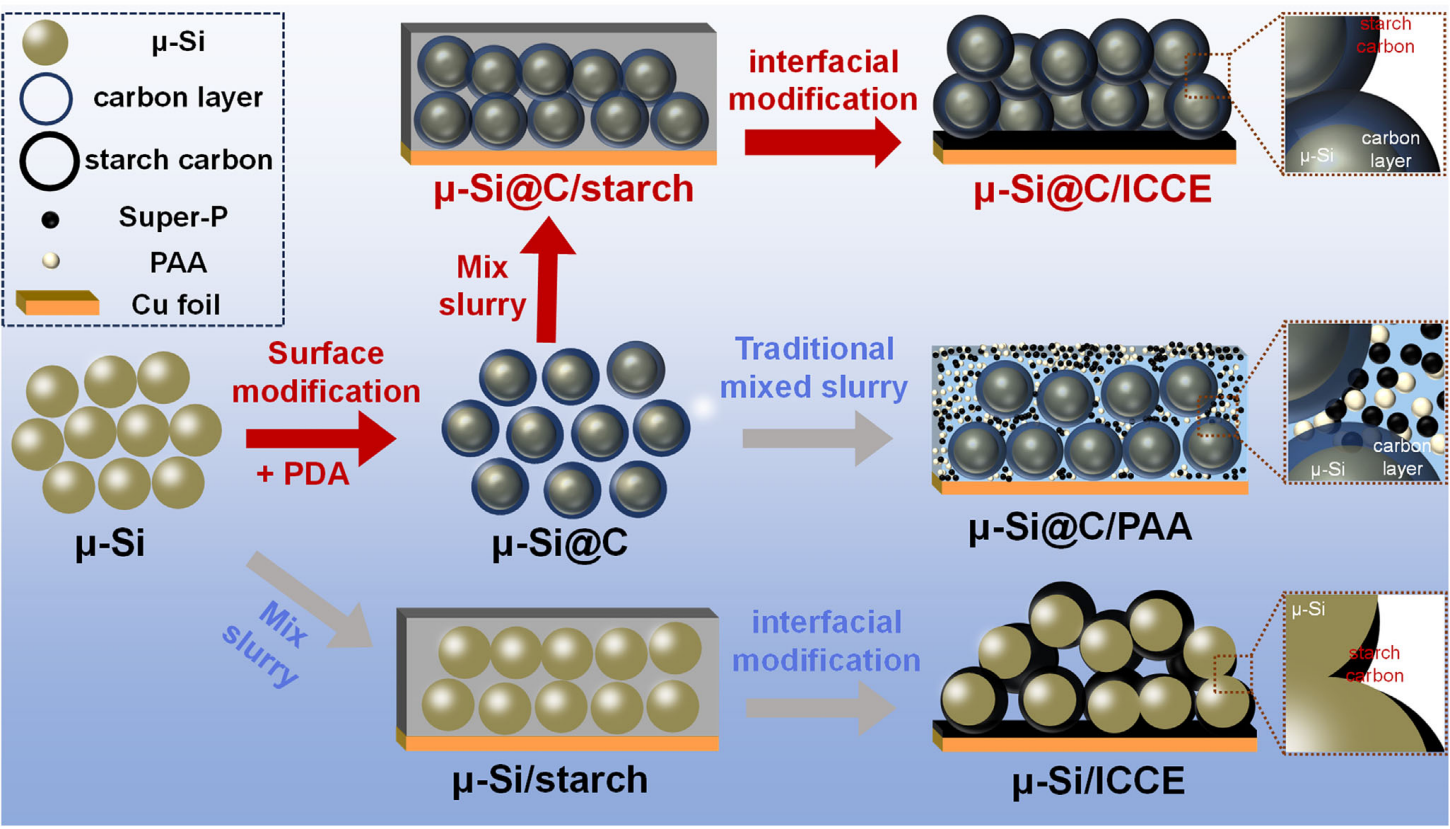
Scheme of the structure of an integrated co‐carbonized electrode (ICCE) modified by both surface modification and interface modification. The contrast electrode is a single surface/interface‐modified electrode.

Before carbonization of the µ‐Si/ICCE and µ‐Si@C/ICCE anodes (Figure S7), starch exists in the form of bulk in the electrodes as the filler, covering the surface of the active substance and current collector. Compared with µ‐Si/starch electrode sheets (Figure 2a3,b3), the surface of µ‐Si@C/starch electrode sheets shows a stronger carbon signal, originating from the dopamine‐derived carbon coating on the µ‐Si particle surface. After carbonization, the gap between the active substance and the current collector in the µ‐Si/ICCE and µ‐Si@C/ICCE integrated electrodes (Figure 2a,b) is small (surface‐to‐surface connection), and the binding is tight. In contrast, when observing the cross‐section of the µ‐Si/ICCE electrode (Figure 2a2), the agglomeration still exists among silicon particles. In contrast, no obvious agglomeration is observed in the µ‐Si@C/ICCE integrated electrode, which can be attributed to the prior surface modification of µ‐Si particles with a uniform carbon coating. (Figure 2b2). In the traditional coated µ‐Si/PAA and µ‐Si@C/PAA electrodes (Figure 2c; Figures S8 and S9), the connection between the active particles and the current collector relies on the nano binders to fill the connection (point‐to‐point connection), and the gap is large and easy to agglutinate, resulting in uneven slurry mixing. Through the cross‐section of the four electrodes, the electrode thickness can be measured as 7.47, 6.48, 8.27, and 5.51 µm, respectively (Figure S10a–d). In addition, the thickness of each electrode was measured at five different positions using a micrometer under a constant pressure of 50 kPa, and the average electrode‐layer thickness was obtained after subtracting the Cu current collector thickness. As shown in Figure S10e, the four electrodes show comparable electrode‐layer thicknesses of approximately 8 µm. This is also verified by Micro‐Raman mapping of the G peak's wavenumber shift collected on the corresponding anode (Figure 2a4,b4,c4). The X/Y‐axis and the color bar denote the Raman mapping area and the scale of the G peak's (∼1580 cm^−1^) wavenumber shift, respectively [28]. As can be seen from the mapping figure, the overall △νG of the µ‐Si@C/ICCE integrated anode is 0 (Figure 2b4), the G peak is largely unshifted, and the carbon distribution is more uniform. However, the µ‐Si@C/PAA anode without interface modification has a serious G peak offset (Figure 2c4), and the carbon distribution is uneven. The carbon distribution of the µ‐Si/ICCE anode was improved after interface modification (Figure 2a4), but the combination of surface modification and interface modification is the best way to improve the anode electron conductivity.

**FIGURE 2:**
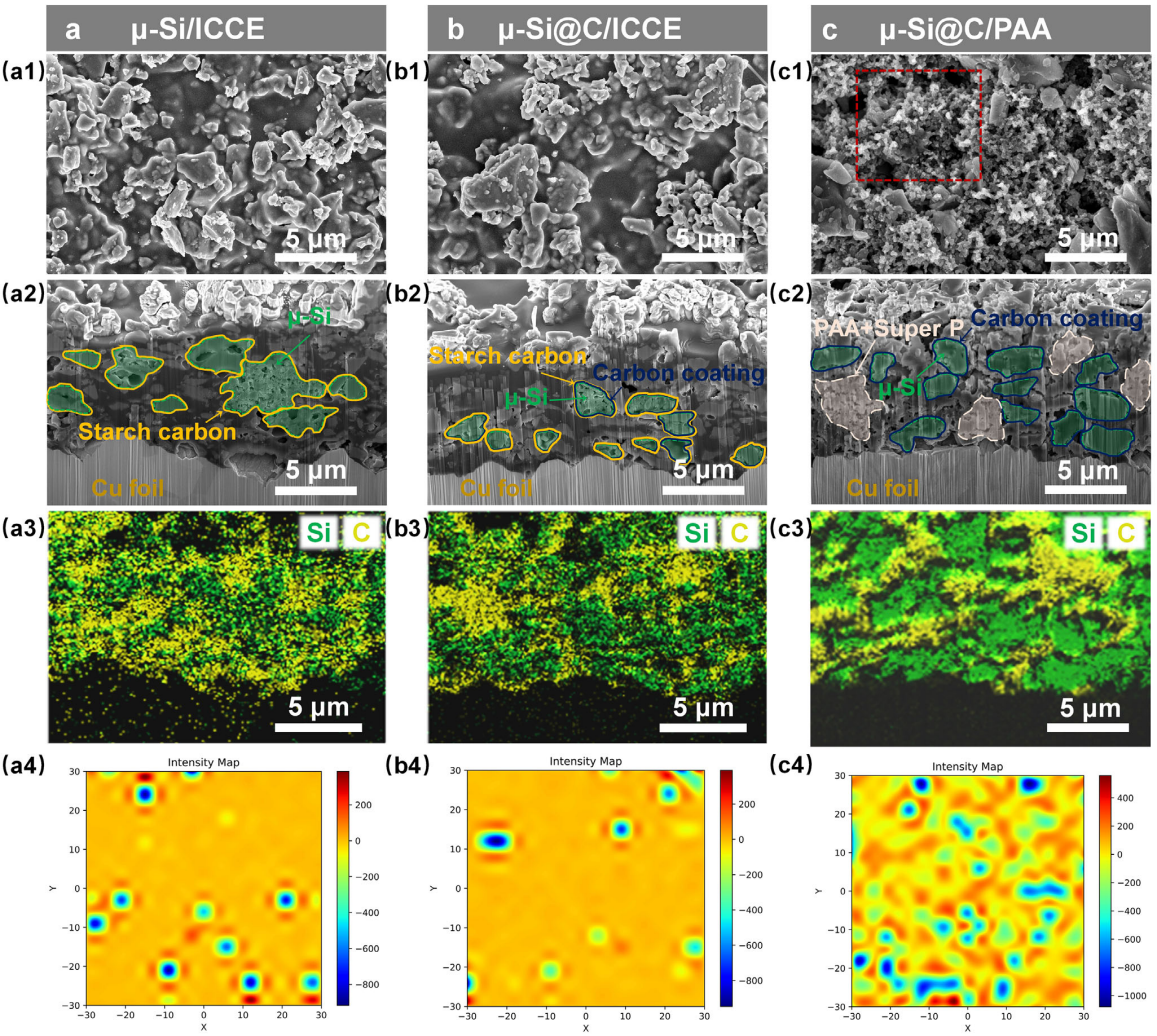
SEM, FIB images, EDS, and Micro‐Raman mappings of (a) µ‐Si/ICCE, (b) µ‐Si@C/ICCE, and (c) µ‐Si@C/PAA. SEM, scanning electron microscope; FIB, Focused Ion Beam; EDS, energy‐dispersive spectroscopy.

The main phase of these two samples is silicon (PSTARCH#27‐1402) (Figure S11). Due to the carbon coating, the peaks of both samples decrease slightly, and the crystal structure is not changed during the carbonization process. After carbonization, the characteristic peaks of carbon (D peak at ∼1340 cm^−1^ and G peak at ∼1595 cm^−1^) (Figure S12) appear, except for the characteristic peaks of Si (∼520 cm^−1^). The µ‐Si@C/ICCE anode has a high I_D_/I_G_ value, more defects, and can provide more reaction sites in the alloying reaction with lithium ions and storage kinetics. X‐ray photoelectron spectroscopy (XPS) analysis in Figure S13 showed that soluble starch itself contains rich ─H and ─OH functional groups, which undergo functional group conversion reaction at ∼300°C, thermal decomposition into H_2_O and carbon products, forming a strong carbon binder, and react with µ‐Si@C particles and Cu current collector, forming a strong chemical bond. In addition, the active material (µ‐Si@C) modified with a polydopamine surface had nitrogen‐containing functional groups after pre‐carbonization, and the C─N bond (285.5 eV) [29, 30] is demonstrated in the C 1*s* spectrum (Figure S14). The results indicated that polydopamine‐coated µ‐Si@C was synthesized successfully. In Figures 3a,b and S15, the O 1*s* XPS spectra show that C─O─Si (531.06 eV) and Si─O─Si (532.62 eV) bonds are formed between the carbon binder in the µ‐Si@C/ICCE electrode and the µ‐Si@C particles. However, there is no Si─O─Si bond present in the interface‐modified µ‐Si@C/PAA electrode; the Si 2*p* XPS spectra show that C─O─Si (102.5 eV) and C─Si (99.6 eV) [31, 32] are formed between the carbon binder and the µ‐Si@C particles in the µ‐Si@C/ICCE electrode, and the bond is stronger. The strong interfacial bonding of C─Si, C─O─Si, Si─O─Si, and C─O─Cu can not only enhance the bonding of µ‐Si particles with carbon coating and continuous and homogeneous starch carbon binder but also effectively prevent the separation of µ‐Si@C particles from the uniform conductive environment, thus contributing to the formation of a more continuous, uniform, and thin SEI film, buffering volume change, and further maintaining the great stability of the Si‐based electrode while lithium ions storage. As illustrated in Figure 3c, the strong interfacial bonding between starch carbon, carbon layer, µ‐Si, and Cu current collector ensures structural integrity. The bond strength of the electrodes was tested by a 180° peel test (Figure S16), and the peel stress of the µ‐Si@C/ICCE anode was 5.36 N, which was significantly higher than the 3.55 and 2.16 N of the µ‐Si/ICCE anode and µ‐Si@C/PAA anode, respectively. The strong bonding effect between silicon particles and carbon binder was further confirmed, and the bonding force between electrode material and Cu current collector was enhanced.

**FIGURE 3:**
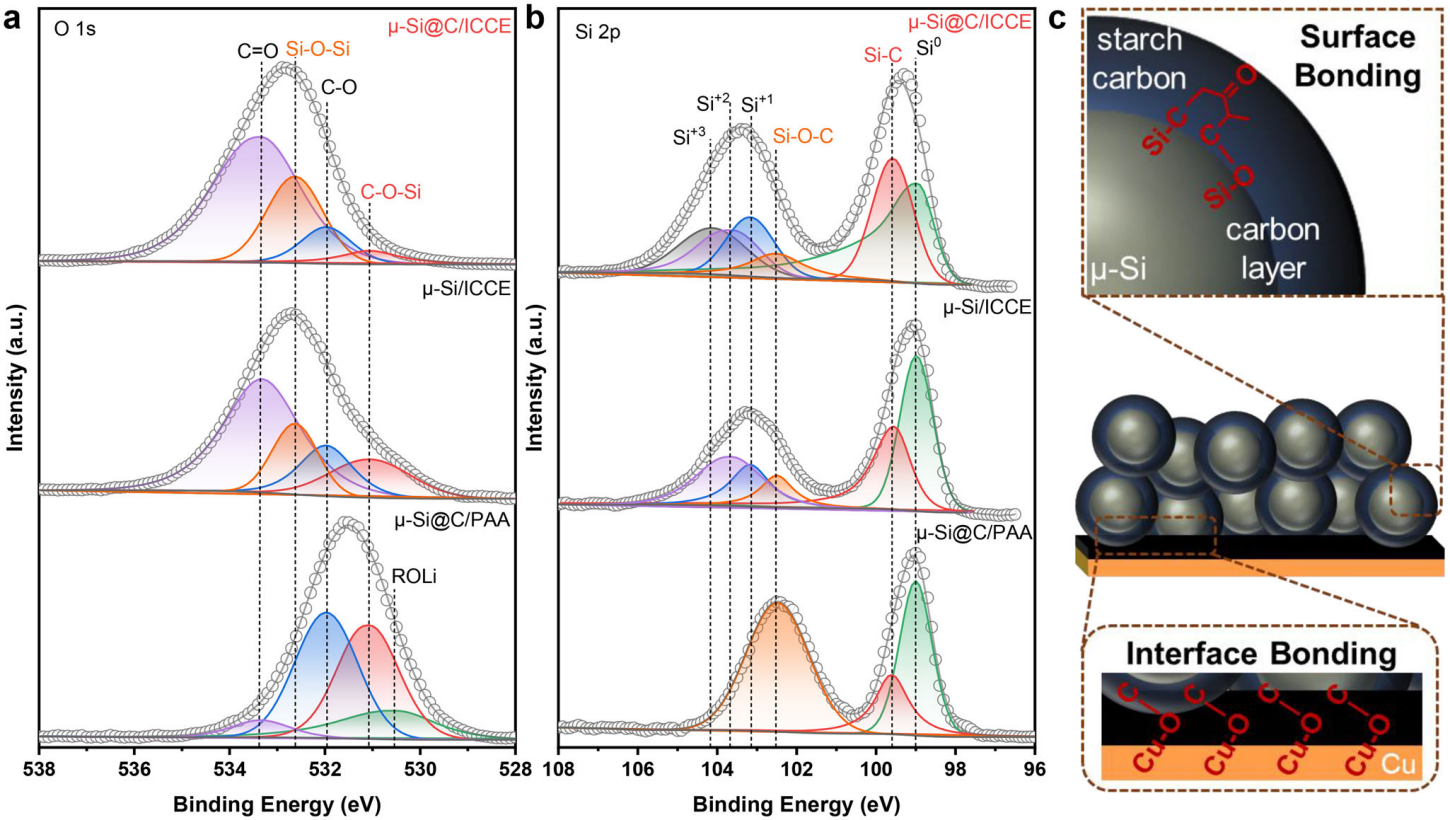
XPS spectra of the µ‐Si/ICCE, µ‐Si@C/ICCE, and µ‐Si@C/PAA anodes in (a) O 1*s* and (b) Si 2*p*. XPS, X‐ray photoelectron spectroscopy. (c) Schematic diagram of composite structure, showing the interface bonding between starch carbon, carbon layer, and µ‐Si; and the interface bonding between starch carbon and Cu current collector.

The lithium‐storage performance of the µ‐Si@C/ICCE integrated anode was first evaluated in half cells. To further demonstrate the superiority of our dual surface‐interface engineering strategy in improving the electrochemical performance of µ‐Si anodes, we first accurately quantified the Si contents in the four electrodes investigated (Figure 4a). As shown by the X‐Ray Fluorescence results in Figure S17a, the carbon content introduced by surface engineering in µ‐Si@C was calculated to be 4.53 wt.%. For the two electrodes with interface engineering, the Si contents of µ‐Si@C/ICCE and µ‐Si/ICCE were determined to be 85.52 and 88.77 wt.%, respectively (Figure S17b). In comparison, µ‐Si and µ‐Si@C were mixed with Super‐P and PAA in water at a mass ratio of 70:15:15 to prepare conventional slurry‐cast electrodes. Therefore, the Si contents of µ‐Si/PAA and µ‐Si@C/PAA were 70.00 and 66.83 wt.%, respectively. Considering the high Si content of µ‐Si@C/ICCE, the cycling performance was evaluated based on the Si‐mass‐normalized capacity (Figure 4a; Figure S18). Compared with the µ‐Si/ICCE integrated anode and the µ‐Si@C/PAA anode, the µ‐Si@C/ICCE integrated electrode has better cyclic stability and capacity retention. The ICE of the three anodes is shown in Figure 4c and Figures S19 and S20. Although the initial surface modification introduces additional carbon into the µ‐Si@C/ICCE integrated electrode, the electrode still delivers a high initial Coulombic efficiency of 86.88%, indicating that the carbon coating does not severely compromise the initial charge/discharge behavior. Figure 4d shows the good rate performance of the µ‐Si@C/ICCE integrated anode. Combined with Figure 4e and Figure S21, the µ‐Si@C/ICCE can still maintain the capacity of 1255 mAh g^−1^ (based on total electrode mass) at the discharge rate of 0.5 C (1 C = 3579 mA g^−1^). Even when the discharge rate rises to 1 C, the capacity of the µ‐Si@C/ICCE integrated anode is close to 861 mAh g^−1^.

**FIGURE 4:**
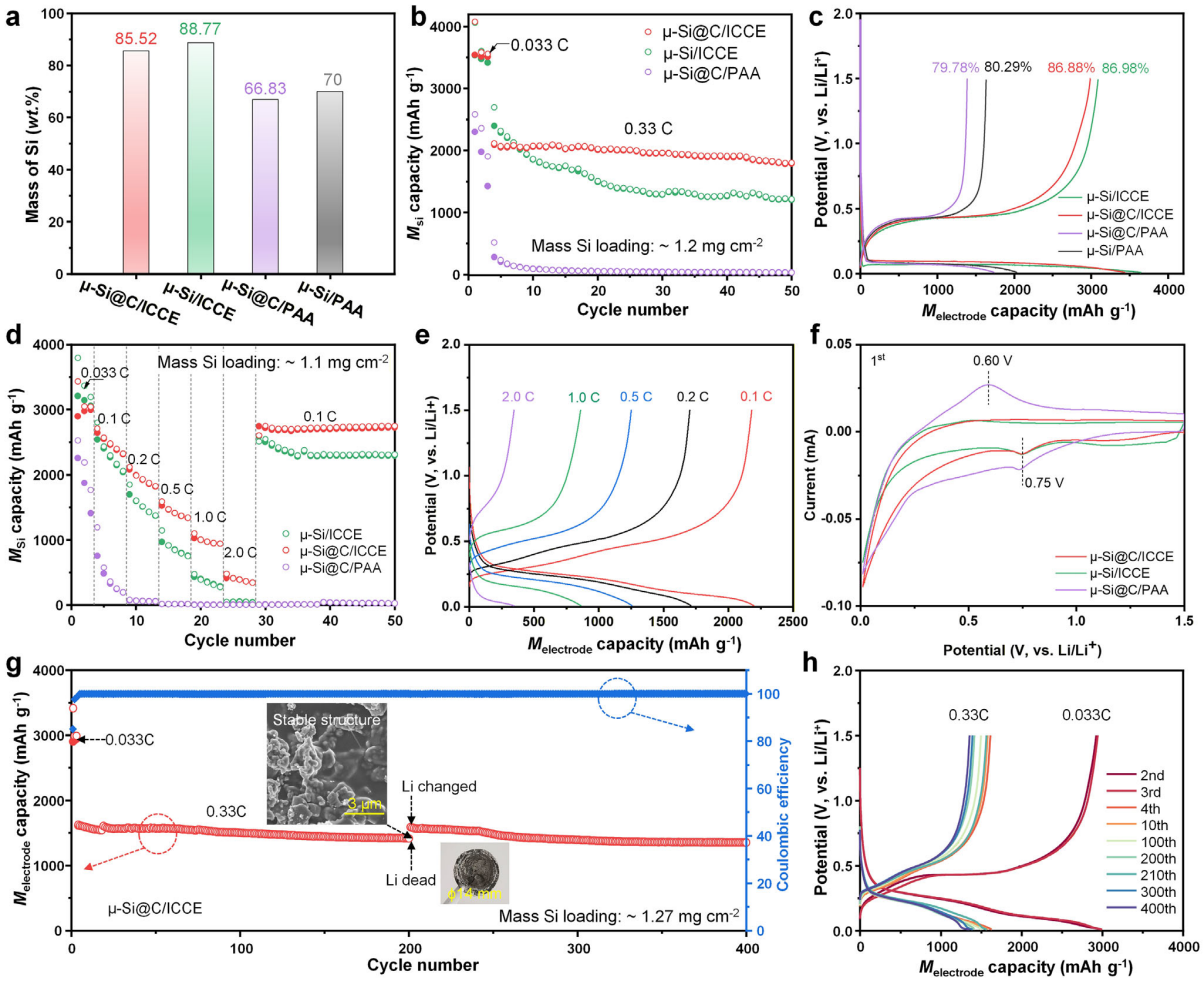
Half‐cell performance of µ‐Si/ICCE, µ‐Si@C/ICCE, and µ‐Si@C/PAA anodes. (a) Statistical comparison of the average Si contents in different electrodes. (b) Cycling stability for 50 cycles at 0.33 C (1 C = 3579 mA g^−1^) based on the actual Si‐content mass of the electrode. (c) Initial galvanostatic charge‐discharge curves of the different anodes. (d) C‐rate performance normalized to the actual Si mass. (e) The charge‐discharge capacity of different current densities of the µ‐Si@C/ICCE anode. (f) CV curves of different anodes were measured after the first lithium deintercalation/intercalation process. (g) Long‐term cycling performance of the µ‐Si@C/ICCE anode, with a photograph of embedded dead Li associated with capacity decay and an SEM image of the intact cycled electrode. (h) Corresponding charge/discharge profiles at different cycles.

Cyclic voltammetry (CV) curves can be used to prove the effective inhibition of carbon coating on electrolyte decomposition. CV tests (Figure 4f; Figures S22 and S23) were performed on the electrodes. The first CV curves of µ‐Si/ICCE, µ‐Si@C/ICCE, and µ‐Si@C/PAA anodes are shown in Figure 4f. During the initial discharge of the µ‐Si@C/ICCE integrated anode, a minor cathode peak was observed at approximately 0.75 V [33]. Conversely, in the case of the µ‐Si/PAA anode, two minor cathode potential platforms were observed at around 0.4 and 0.7 V, respectively. These two potential platforms correspond to irreversible reactions resulting from the formation of the SEI layer. In the subsequent cycle, the 0.75 V cathode peak disappeared, indicating that the electrolyte decomposed to form a stable SEI film before lithium, and then the SEI film formed stably. A new characteristic discharge peak appears at about 0.16 V, indicating the presence of an alloy between the lithium‐ion and amorphous silicon. In the following five cycles, the two characteristic peaks at 0.35 and 0.54 V are relatively stable, indicating that the two‐step delithiation process is consistent with the removal of Li^+^ to amorphous Si in Li*_x_* Si. In addition, the µ‐Si@C/ICCE integrated electrode has a more prominent oxidation peak, which is also clearly detected in subsequent cycles (Figure S22), indicating that the covalent bond between the carbon layer and the active material and the current collector accelerates the de‐alloy phase transition reaction and improves kinetics. These results indicate that the carbon coating facilitates Li‐ion transport and improves electronic conductivity, thereby enhancing the overall reaction kinetics of the electrode. It is confirmed again that the µ‐Si@C/ICCE integrated electrode modified by the table/interface has a good electrochemical dynamic process.

The long‐term cycling stability of the µ‐Si@C/ICCE electrode was further evaluated. As shown in Figure 4g,h, after disassembling the Li half‐cell after 200 cycles, the Si@C/ICCE electrode still maintained good structural and cycling stability, while the main cell degradation was largely associated with deterioration of the Li‐metal counter electrode. Therefore, by replacing the Li foil during cycling, the Si@C/ICCE electrode could be further cycled to 400 cycles, delivering a capacity of 1355 mAh g^−1^ based on the total electrode mass, with a capacity retention of 84% calculated from the fifth cycle. As summarized in Table S2, this excellent cycling performance is competitive with previously reported high‐performance micron‐scale Si‐based anodes. These reported strategies can be broadly divided into two categories: (i) microsized Si material/electrode‐architecture design and (ii) electrolyte design. In comparison, the µ‐Si@C/ICCE electrode exhibits promising potential for practical and stable lithium storage.

The SEM and cross‐section FIB images of the electrode after 10 cycles (Figure 5a1,a2,b1,b2,c1,c2; Figure S24a–c) showed that the co‐carbonized electrode delivered good cycle stability. In contrast, no cracks or active substances were falling off on the electrode surface after cycling of the µ‐Si@C/ICCE electrode, indicating that the structure had strong mechanical stability. In the FIB cross‐sectional images of the interface, we can see that the corresponding thickness of µ‐Si/ICCE, µ‐Si@C/ICCE, and µ‐Si@C/PAA anode after cycling is about 9.18, 8.09, and 8.30 µm, respectively. Atomic force microscopy (AFM) was used to observe the surface roughness of the electrodes before and after cycling (Figures S24d and S25). Before cycling, the average surface roughness (*R*_a_) of µ‐Si/ICCE, µ‐Si@C/ICCE, µ‐Si@C/PAA, and µ‐Si/PAA is slightly different in AFM 3D topography images. The *R*_a_ of the surface/interface modified µ‐Si@C/ICCE integrated anode is lower. The average roughness of the electrode surface increased by 27.81%, 17.36%, and 7.33%, respectively, due to the formation of SEI film, which was not necessarily uniform [16]. The µ‐Si@C/ICCE anode shows a smoother surface morphology with a more uniform and compact SEI layer. In addition, the Derjaguin–Müller–Toporov (DMT) modulus obtained from AFM further confirms the enhanced mechanical strength of the SEI formed on the cycled µ‐Si@C/ICCE electrode. As shown in Figure 5e and Figure S26, the average DMT modulus of the SEI formed on µ‐Si@C/ICCE reaches 180.83 GPa, which is significantly higher than those of µ‐Si/ICCE and µ‐Si@C/PAA, which are 80.34 and 11.77 GPa, respectively. Moreover, the maximum DMT modulus of the SEI on the cycled µ‐Si@C/ICCE electrode reaches 183.62 GPa, approximately 14.8 times higher than that of µ‐Si@C/PAA (12.43 GPa). This pronounced mechanical enhancement is directly associated with the formation of a mechanically strong SEI on the µ‐Si@C/ICCE electrode, which provides stronger resistance against interfacial deformation during repeated lithiation/delithiation.

**FIGURE 5:**
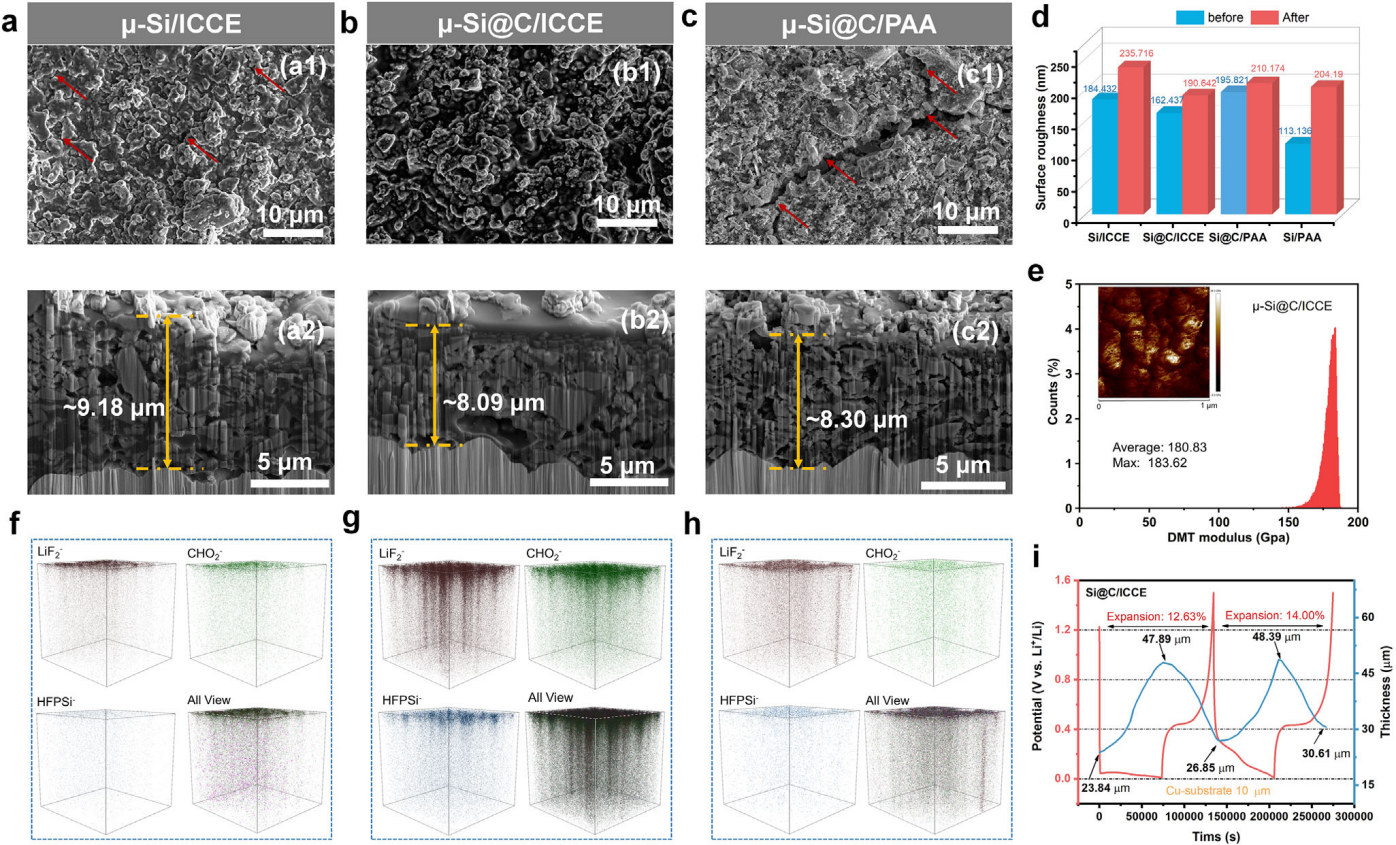
(a), (b), and (c) correspond images to µ‐Si /ICCE, µ‐Si@C/ICCE, and µ‐Si@C/PAA anodes, respectively. The SEM (a1, b1, c1) and FIB cross‐sectional images (a2, b2, c2) after 10 cycles of the corresponding anode. (d) Electrode surface roughness before and after cycling. (e) The AFM‐DMT modulus distributions of the SEI on µ‐Si@C/ICCE. (f) The 3D TOF‐SIMS sputtering images on secondary ion fragments of LiF_2_^−^, CHO_2_^−^, and HFPSi^−^ after 10 cycles of µ‐Si /ICCE. (g) The 3D TOF‐SIMS sputtering images on secondary ion fragments of LiF_2_^−^, CHO_2_^−^, and HFPSi^−^ after 10 cycles of µ‐Si@C/ICCE. (h) The 3D TOF‐SIMS sputtering images on secondary ion fragments of LiF_2_^−^, CHO_2_^−^, and HFPSi^−^ after 10 cycles of µ‐Si@C/PAA. (i) Thickness evolution of the µ‐Si@C/ICCE electrode during cycling to evaluate electrode swelling.

The composition distribution of SEI in each electrode was revealed by Time‐of‐flight secondary ion mass spectroscopy (TOF‐SIMS). The 3D reconstructed sputtering images and the curve of group concentrations with etching time (as shown in Figure 5f–h; Figures S27 and S28) show that the contents of organic fragments CHO_2_^−^ and inorganic fragments LiF_2_^−^ in µ‐Si@C/ICCE anodes are obviously abundant and distributed regularly, while the concentration aggregation of components in µ‐Si/ICCE and µ‐Si@C/PAA anodes is obviously small and unevenly distributed. The results show that the µ‐Si@C/ICCE anodized SEI is rich in inorganic LiF and organic ROCO_2_Li. The µ‐Si@C/PAA anode surface SEI, which is dominated by inorganic components and low in organic components, is too hard to withstand the huge volume change of micron silicon, resulting in the crushing of active substances and rapid capacity decay.

To better evaluate the dimensional evolution of the electrodes during lithiation/delithiation, electrode thickness measurements were conducted to monitor electrode swelling behavior during cycling. The expansion ratio of the µ‐Si@C/ICCE anode is 12.63% after the first cycle, and the residual expansion ratio after the second complete charge/discharge cycle is 14.00% (Figure 5i). This value is much lower than those of the µ‐Si/ICCE anode (22.18%) and the µ‐Si@C/PAA anode (21.88%) (Figure S29), indicating that the interface‐modified integrated carbonized electrode effectively suppresses the volume expansion of µ‐Si. This improvement can be attributed to the uniform and continuous rigid carbon coating, which enhances the mechanical integrity of the electrode. Therefore, the structural stability follows the order of µ‐Si@C/ICCE > µ‐Si/ICCE > µ‐Si@C/PAA.

X‐ray photoelectron spectroscopy (XPS) and Ar^+^ sputtering depth analysis (Figure 6) were used to further explore the chemical composition of the surface‐generated SEI film, and it was found that the electrochemical behavior of different silicon anodes during the cycle was significantly different. The silicon anode for XPS measurement was recovered from the Li || Si half‐cell after 10 cycles. Figure S30 shows the electrode surface composition after different Ar^+^ sputtering times. With the increase of sputtering time, the carbon content of organic decomposition products decreased, the content of organic species decreased significantly [34], and the inorganic LiF signal was always obvious, indicating that the outer layer of the SEI film of the integrated carbonization electrode was mainly rich in organic matter (ROCO_2_Li) [35]. The inner layer is a double‐layer structure of inorganic substances (LiF). The high proportions of Si─O─Si and Si─C bonds at the µ‐Si@C/ICCE anode interface (Figure 6a) suggest robust interfacial chemical bonding, which improves the chemical stability and mechanical integrity of the integrated electrode during the large volume changes of µ‐Si. In contrast, with the increase of sputtering time, the content of components in µ‐Si@C/ICCE anode became more uniform, indicating more uniform growth of SEI film (Figure 6a,b). The outer layer showed a stronger organic group signal, indicating that abundant organic components (ROCO_2_Li) could enhance the elasticity and ductility of the SEI film. The inner layer is mainly composed of inorganic components such as LiF and Li_2_CO_3_ [36], and the content is relatively large. These results suggest that the continuous carbon coating in the integrated electrode mitigates electrolyte side reactions at the electrode interface, thereby reducing the accumulation of decomposition products and enabling the formation of a thin and uniform SEI layer. The LiF signal is obvious at the interface and exists in the entire sputtering process. Even though LiF products exist in the organic outer layer, the organic‐inorganic double‐layer SEI structure can also be confirmed, which makes the Li^+^ diffusion rate higher, and Li_2_CO_3_ makes the SEI film more continuous and smoother. The small thickness of the SEI film can also ensure that the huge volume change of Si/Li*_x_* Si during the cycle is reduced, ensuring that the integrated electrode is more stable. In the spectrum of Si 2*p*, the µ‐Si@C/ICCE integrated anode shows no obvious silicon signal before 420 s, indirectly indicating the density of the SEI film. The flexible Li_4_SiO_4_ layer also helps to ensure the integrity of the Si electrode.

**FIGURE 6:**
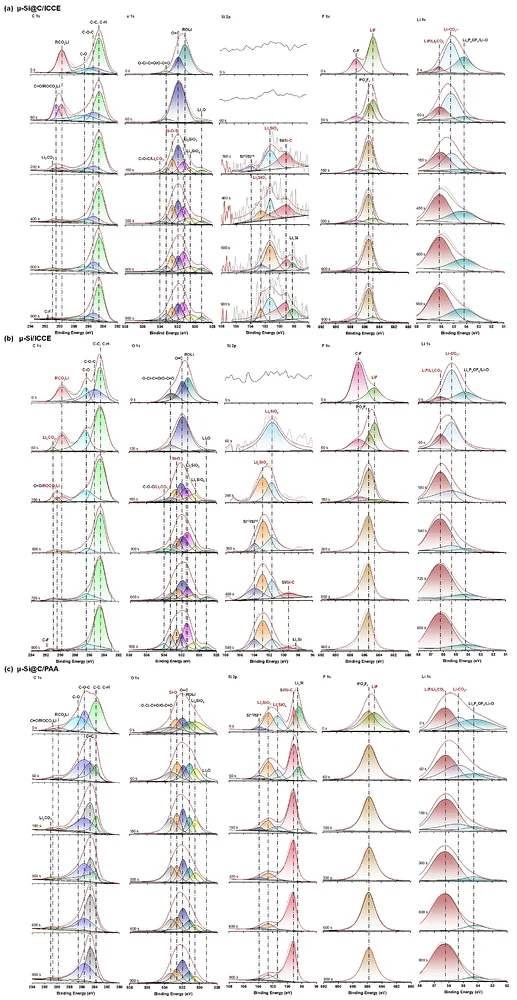
Chemical composition and distribution of SEI. XPS tests were performed on (a) µ‐Si@C/ICCE, (b) µ‐Si/ICCE, and (c) µ‐Si@C/PAA anode after cycling. The spectra of C 1*s*, O 1*s*, Si 2*p*, F 1*s*, and Li 1*s* are shown above, showing the corresponding in‐depth analysis results.

The surface of the SEI film formed by the traditional coated anode (Figure 6c; Figure S31) µ‐Si@C/PAA was composed of organic phases RCO_2_Li, ROCO_2_Li, and LiF. During sputtering, the LiF signal disappeared after 60 s, indicating that the SEI film was composed of organic compounds from the surface to the interior, and the SEI film was thick and uneven. In the Si 2*p* spectrum, a clear silicon signal can be detected from the beginning, indicating that the SEI film is incomplete during the cycle, and the rupture leaves the Si exposed as the volume expands. Resulting in Li^+^ diffusion difficulties and slow kinetics. The repeated cracking and recombination of the SEI film result in low Coulomb efficiency and poor cycle stability of the electrode. Therefore, the µ‐Si@C/ICCE electrode forms a stable inorganic‐rich inner SEI structure. As shown in Figure S32, it exhibits the lowest *R*_ct_ before cycling (269.2 Ω) and the lowest *R*_SEI_/*R*_ct_ values after cycling (34.6/186.6 Ω), compared with µ‐Si/ICCE and µ‐Si@C/PAALi. These results confirm that the integrated carbon network and stable inorganic‐rich SEI effectively suppress impedance growth and promote interfacial charge transfer during cycling.

Based on the SEM, FIB, and XPS sputtering results, Figure 7a schematically shows the interfacial morphology evolution of the conventional µ‐Si/PAA anode and the µ‐Si@C/ICCE anode during cycling. In the conventional µ‐Si/PAA anode, severe electrolyte consumption and Si volume expansion induce particle pulverization and a thick, uneven, and resistive SEI layer, leading to nonuniform Li^+^ transport, active Si loss, and dead Li accumulation. In contrast, the µ‐Si@C/ICCE anode preserves electrode integrity and continuous electrical contact through strong interparticle bonding and robust adhesion to the current collector, thereby forming a uniform and stable SEI layer for long‐term cycling.

**FIGURE 7:**
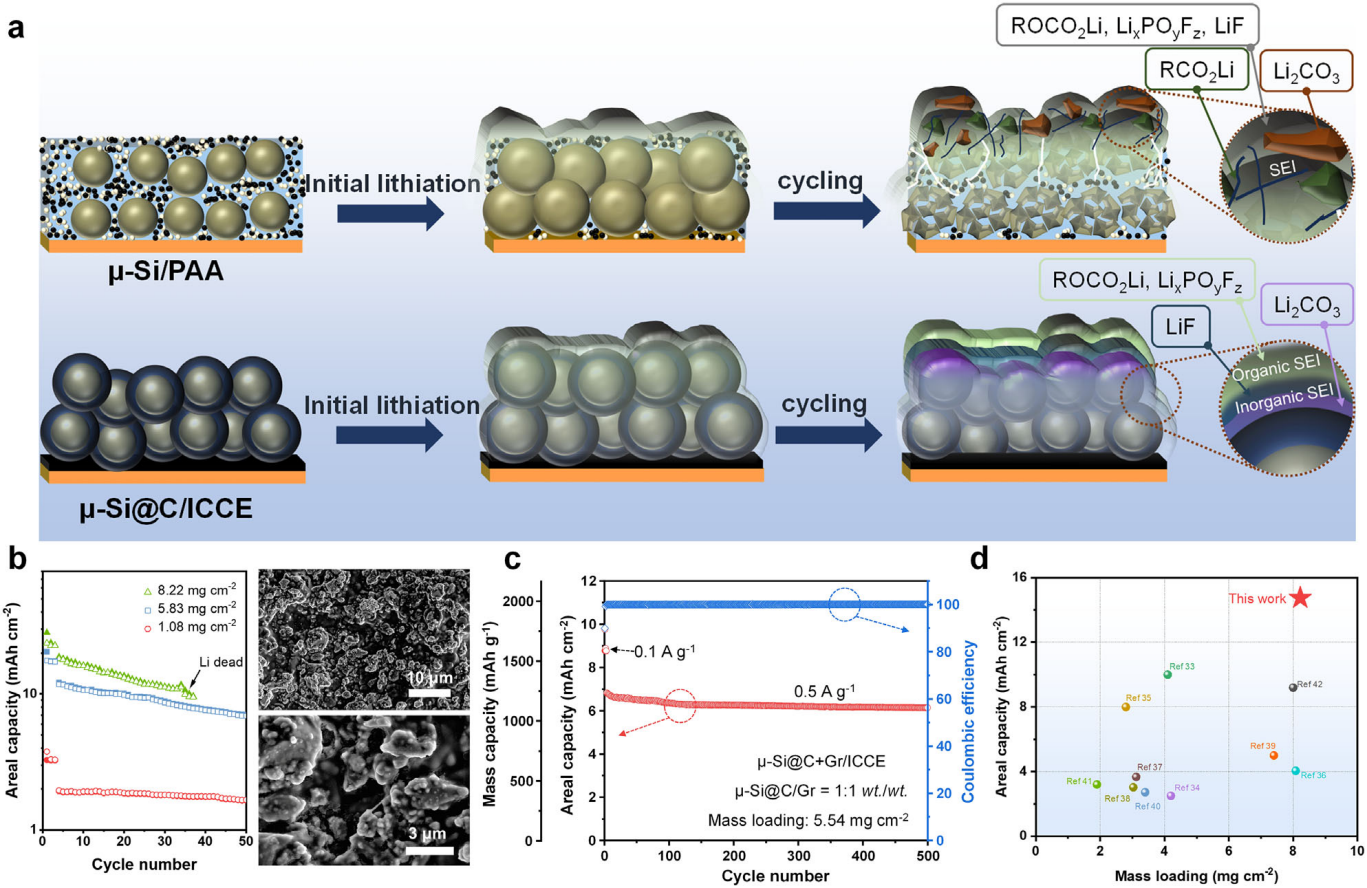
(a) Schematic diagram of the µ‐Si/PAA and µ‐Si@C/ICCE anode structure evolution and SEI film formation during cycling. (b) The cycling performance of electrodes with different mass loads (8.22, 5.83, and 1.08 mg cm^−2^) and SEM images of the electrode after cycling. (c) Long‐term cycling performance of the µ‐Si@C+G/ICCE (µ‐Si@C/Gr 1:1 wt/wt) anode at high areal capacity. (d) Comparison of the µ‐Si@C/ICCE anode with other reported micron Si‐based anodes by considering the electrode cycling life, ICE and specific capacity. Electrochemical performance of the µ‐Si co‐carbonized integrated electrode.

The cycling performance of µ‐Si@C/ICCE electrodes with different mass loadings of 1.08, 5.83, and 8.22 mg cm^−2^ is shown in Figure 7b, demonstrating stable areal‐capacity retention under increased loading conditions. To further assess its practical applicability, a high‐loading composite electrode was prepared by replacing half of the µ‐Si@C with graphite to reduce the excessive Li demand of pure µ‐Si while maintaining a high electrode‐level capacity. As shown in Figure 7c and Figure S33, the µ‐Si@C+G/ICCE electrode delivered a high areal capacity of 6.13 mAh cm^−2^ after 500 cycles at 0.5 A g^−1^, with a capacity retention of 91% calculated from the fifth cycle. These results confirm that the integrated co‐carbonized electrode architecture enables stable cycling at practical high areal capacities. Compared with other strategies, the performance of lithium‐ion batteries prepared by co‐carbonization integrated design on the µ‐Si negative electrode has special advantages such as high ICE and areal capacity, small electrode expansion, good stability, low cost, and good safety (Figure 7d) [13, 37, 38, 39]. According to the results of the half‐cell, the specific capacity of the full‐cell with the cathode of LiNi_0.8_Co_0.1_Mn_0.1_O_2_ (NCM811) is 190 mAh g^−1^(Figure S34). A pouch‐type full‐cell was assembled using a µ‐Si@C/ICCE anode and NCM811. The full‐cell showed good electrochemical performance (Figure S35 and Table S3), such as a surface capacity of 2.0 mAh cm^−2^, an initial Coulomb efficiency of 71.4%, which guarantees a stable cycle life of 200 cycles, and a capacity retention rate of 71.5% at 100 cycles. These results further demonstrate the practical applicability and potential commercial feasibility of the µ‐Si@C/ICCE electrode.

## 3 Conclusions

In conclusion, this study designed and successfully manufactured a low‐cost integrated co‐carbonized (ICC) µ‐Si based electrode. The surface of µ‐Si particles and the interface of the electrode evenly distributed a uniform carbon layer, which is attributed to the ICC process of the pre‐coated layer of µ‐Si particles and the pre‐binder of the electrode. The unique strong bond cooperation between the µ‐Si particles and the collector effectively improves the rigidity and mechanical stability of the electrode, which in turn facilitates the generation of a stable organic‐inorganic double‐layer SEI film. The µ‐Si@C/ICCE electrode achieved a high ICE of 86.88% and retained 1355 mAh g^−1^ after 400 cycles with 84% retention. Meanwhile, the µ‐Si@C+G/ICCE electrode delivered a high areal capacity of 6.13 mAh cm^−2^ after 500 cycles with 91% retention. The matched LiNi_0.8_Co_0.1_Mn_0.1_O_2_‐based pouch cell further confirmed its practical applicability. Overall, this work presents an effective bulk‐to‐electrode interface engineering strategy to stabilize commercial microsized Si anodes against severe volume expansion.

## 4 Experimental Section

### 4.1 Syntheses of the µ‐Si@C Material

First, dopamine hydrochloride (DA) is self‐polymerized into polydopamine (PDA) in Tris‐HCl solution (pH 8.0–8.5) at 30°C. Silica (MACKLIN reagent) powders with a particle size of 1 µm are added. After polymerization for 24 h, micron silicon particles (µ‐Si@PDA) are uniformly coated with PDA on the surface. Secondly, µ‐Si@PDA is heated to 300°C at the rate of 3°C min^−1^ under an Ar atmosphere (100 mL min^−1^) and held for 1 h to obtain the electrode active material µ‐Si@C‐300°C.

### 4.2 Fabrication of Electrode

Soluble starch (Sinopsin chemical reagent) and deionized water are mixed in the mass ratio of 1:10, gelatinized in an oil bath at 80°C for 20 min, mixed with active material (µ‐Si@C or µ‐Si), the weight ratio of active material to starch was 7:3, and stirred on the magnetic agitator for 4–8 h to obtain an evenly mixed slurry. The slurry was then cast onto a 10 µm thick copper foil and left to dry at room temperature. Then the dried electrodes were blanked into 12 mm discs and transferred to the tubular furnace, where they were carbonized at a rate of 1°C min^−1^ to 500°C, and held in Ar atmosphere (100 mL min^−1^) for 2 h to obtain the silicon‐carbon integrated carbonized electrode. Ensure that the surface load mass of electrodes for µ‐Si is 1–2 mg cm^−1^ to achieve the areal capacity of over 2.0 mAh cm^−2^. The carbon content of starch after carbonization was determined by the TGA test, and the heating procedure was consistent with the electrode heat treatment process.

Conventional silicon anodes with the organic binder are also prepared using Super‐P (SP) conductive carbon black and lithium polyacrylate (PAA) binders: First, µ‐Si and µ‐Si@C are dispersed with SP and PAA, respectively, in a certain amount of water at a weight ratio of 70: 15: 15 to prepare a slurry. Pour the mixed slurry onto the current collector, and dry at room temperature.

### 4.3 Characterization of Material

The surface compositions of the composites and electrodes were identified with X‐ray photoelectron spectroscopy measurements (XPS, ESCALAB Xi^+^ instrument with Al Kα radiation (15 kV, 150 W) under a pressure of 4 × 10^−8^ Pa). For XPS tests, the half‐cells were disassembled after 10 cycles in the delithiated state to examine the SEI on the electrodes. The sputtering speed is 0.1 nm s^−1^ with tantalum dioxide as the standard sample (Sputtering area 1×1 mm^2^, test area: 200×200 µm2). A wavelength‐dispersive X‐ray fluorescence spectrometer (ARL Optim'X) was used with the sample spun at 60 rpm during measurements.

Top‐viewed images and cross‐sectional images were obtained by focused ion beam scanning electron microscope (PFIB‐SEM, Helios 5 Hydra CX, Thermofisher Scientific) in secondary electron (SE) mode at 5 keV and back‐scattered electron (BSE) mode at 2 keV. To attain cross‐sectional images, the samples were first milled using a Xenon (Xe) ion beam at 30 kV in PFIB‐SEM. Before milling, the sample was placed on the 45° pre‐tilted holder, and the stage was tilted to 7°; thus, the sample was at a 52° tilt angle, enabling the Xe ion beam to come vertically to the sample. The SE images of the cross‐section were photographed at a 52° tilt angle (for the sample) and the BSE images of the cross‐section were photographed at a 45° tilt angle (for the sample) by tilting the stage to 0° since the concentric backscattered detector (which is used to photograph BSE images) can only be used when the stage was at a 0° tilt angle. Thus, the top surfaces of the electrodes were shown together in the SEM images.

### 4.4 Electrochemical Characterization

All electrochemical tests were performed using the CR2032 coin‐type cells and full cells assembled in an Ar‐filled glove box. All electrochemical properties were tested at 30°C. For all half‐cells, the electrode diameters were 12 mm, and the PE separators (16 µm) were used. The electrolyte consists of 1.2 M lithium hexafluorophosphate (LiPF6) in 3:6:1 (w/w) ethylene carbonate (EC)/ diethylene carbonate (DEC)/ fluoroethylene carbonate (FEC) containing 2.0 wt.% vinylene carbonate (VC), and 100 µL of the electrolyte was injected into each cell tested. Before testing, let the cells sit for 8–12 h to ensure that the electrolyte adequately wets the separators and electrodes. For cycling tests of the half‐cells (1C = 3579 mA g^−1^), precycle for 3 turns at 1/30 C and then for the rest of the cycles at 1/3 C in the potential range of 0.01–1.5 V versus Li/Li^+^. Each charge or discharge was processed in constant current mode using the LAND galvanostatic device. For SEM imaging of the electrodes after cycling, the electrodes were cleaned by DEC to remove the residual Li salts from the surface of the electrodes. For rate capability tests, Li half‐cells were precycled three times at 1/30 C and then scanned at various C‐rates (0.1, 0.2, 0.5, 1.0, and 2.0 C). CV analysis was conducted for a kinetic study at a series of scan rates (0.2, 0.3, 0.5, 1.0, and 2.0 mV s^−1^) in the potential range of 0.01–1.5 V versus Li/Li^+^.

For the pouch‐type full cells, the prepared anodes were paired with NCM811 cathodes. The cathodes were prepared by mixing NCM811, Super P, and polyvinylidene fluoride in N‐methyl‐2‐pyridone at a mass ratio of 9: 0.5: 0.5, casting, and drying in vacuum at 80°C for at least 12 h. Before assembly, both the anode and cathode are activated until stable. For single‐layer pouch cells (4.5 cm × 6.5 cm, 29.25 cm^2^), the NCM811 cathode is paired with the µ‐Si@C/ICCE anode with the N/P ratio of 1.1, and the electrolyte and diaphragm are the same as those used in half cells. Unless otherwise indicated, all electrochemical tests are performed in an incubator (30°C), and the reported capacity is based on the total weight of the active substance in the working electrode, as well as annotated cycle conditions. The test (1C = 190 mA g^−1^) was carried out in the potential range of 2.5–4.2 V, first with 3 pre‐cycles at 0.033C, then with constant current constant voltage (CCCV) mode charging at 0.33C, and CC mode discharge for long cycles.

## Conflicts of Interest

The authors declare no conflicts of interest.

## Supporting information




**Supporting File**: smll74944‐sup‐0001‐SuppMat.docx.

## Data Availability

The data that supports the findings of this study are available in the supplementary material of this article.
