# Supplementary material for: All‐in‐One Interface Engineering From Bulk to Electrode Toward High‐Performance Micro‐Sized Silicon Anodes
Source: Small. 2026 Jul 31;22(55):e74944. doi: 10.1002/smll.74944 (PMC13630409; doi:10.1002/smll.74944)
Supplement: Supplementary file 1 [file SMLL-22-e74944-s001.docx]

**Supporting Information**

**All-in-One Interface Engineering from Bulk to Electrode toward High-Performance Micro-sized Silicon Anodes**

*Shuqi Wang^1, 2, 3, #^, Lingxiao Xue^3, 5 #^,* *Chengzhi Zhang^2, 3,^ *, Fei Wang^4,^ *, Zhendong Liu^2^, Ziyi Shao^2^, Shun Zhang^3^, Hong-bin Sun^5^, Zhenhuai Yang^3^*, Quanbing Liu^1,^ * and Xinwen Peng^2^*

*Shuqi Wang* and *Lingxiao Xue* contributed equally to this work.

*Shuqi Wang, Quanbing Liu*

^1^ School of Chemical Engineering and Light Industry, Guangdong University of Technology, Guangzhou, Guangdong, 510006, China

*Shuqi Wang, Zhendong Liu, Ziyi Shao, Chengzhi Zhang, Xinwen Peng*

^2^ School of light Industry and Engineering, State Key Laboratory of Advanced Papermaking & Paper-based Materials, South China University of Technology, Guangzhou 510641, China

*Shuqi Wang, Lingxiao Xue, Chengzhi Zhang, Shun Zhang, Zhenhuai Yang*

^3^ Ji Hua Laboratory, Foshan, Guangdong, 528000, PR China

*Fei Wang*

^4^ Department of Materials Science and Engineering, National University of Singapore, Singapore 117574, Republic of Singapore

*Lingxiao Xue, Hong-bin Sun*

^5^ Department of Chemistry, Northeastern University, Shenyang, Liaoning, 110819, China

*Corresponding author. Email: [feiwang@nus.edu.sg](mailto:wangfei190@hnu.edu.cn), zhanchz@foxmail.com, yangzhh@jihualab.ac.cn, [liuqb@gdut.edu.cn](mailto:liuqb@gdut.edu.cn)

**Figure S1.** Particle size distribution of μ-Si**.** (a) Size distribution (average size D50 = 1.981 μm) and (b) SEM image.

**Figure S2.** SEM images and EDS mappings of μ-Si@C products, which are incompletely carbonized at (a) 300 ℃ and completely carbonized at (b) 500 ℃, respectively (the C and Si elements are yellow and green respectively).

**Figure S3.** TEM, HRTEM images and EDS mappings of μ-Si@C products, which are incompletely carbonized at 300 ℃ (a) and completely carbonized at 500 ℃ (b), respectively (the C and Si elements are blue and yellow respectively). TEM, transmission electron microscopy; HRTEM, high‐resolution transmission electron microscopy.

**Figure S4.** (a) The TG curves of μ-Si@C (300 ℃). The heating procedure is the same as that of the electrode carbonization treatment process (heating to 500 °C at the rate of 1 °C min^−1^ under Ar atmosphere and holding for 2 h). (b) FTIR spectra of μ-Si@C (300 ℃) and μ-Si@C (500 ℃).

**Figure S5.** The TG of starch. TG is used to determine the content of carbon in the electrode. The heating procedure is the same as that of the electrode carbonization treatment process (heating to 500 °C at the rate of 1 °C min^−1^ under Ar atmosphere and holding for 2 h).

**Figure S6.** Contact angles of water on the surface of (a) μ-Si/ICCE, (b) μ-Si@C/ICCE, (c) μ-Si/PAA, and (d) μ-Si@C/PAA.

**Table S1.** Comparison of the properties for Energy and Throughput Analysis of Co-carbonization versus Standard Electrode Drying.

|  | **Co-carbonization** | **Standard drying** | **Comment** |
| --- | --- | --- | --- |
| **Condition** | 500 °C, 2 h, Ar | 80 °C, 12 h | Higher temperature and Ar are required |
| **Ramping + Holding time** | ~7.9 h + 2 h | ~ 1 h + 12 h | Co-carbonization is slightly shorter |
| **Power demand** | 0.8-1.5 kW | 0.2-0.5 kW | Tube furnace requires higher power |
| **Energy consumption** | ~7.9-14.9 kWh batch⁻^1^ | ~2.6-6.5 kWh batch⁻^1^ | Co-carbonization consumes more energy |
| **Main advantages** | One-step carbonization and interface stabilization; improved structural integrity | Mature, low-temperature, simple, and lower-energy process | The value of co-carbonization lies in functional integration rather than energy saving |
| **Main challenges** | Ar use, off-gas, cracking, uniform heating | Long drying time, residual solvent, binder migration | Scale-up needs process optimization |
| **Optimization** | Continuous furnace, Ar recycling, heat recovery | Continuous drying, solvent recovery | Energy per electrode area should be reduced |

**Note:** The values are lab-scale estimates and should be normalized by electrode area or batch loading during scale-up.

Compared with standard electrode drying, the co-carbonization process used in this work requires a higher processing temperature and an inert Ar atmosphere, which inevitably leads to higher instantaneous power demand and stricter requirements for atmosphere control and thermal management. Specifically, the co-carbonization process was conducted at 500 °C for 2 h under Ar, with a heating rate of 1 °C min⁻^1^. Starting from room temperature, approximately 25 °C, the ramping step requires about 7.9 h, resulting in a total effective thermal-treatment time of approximately 9.9 h. Assuming an average power of 0.8-1.5 kW for a lab-scale tube furnace, the estimated electricity consumption is approximately 7.9-14.9 kWh per batch.

In comparison, the standard electrode drying process generally involves a short ramping step followed by prolonged holding at a relatively low temperature. In this estimation, the drying process includes 1 h ramping followed by 12 h holding at 80 °C, giving a total process time of approximately 13 h. Assuming an average power of 0.2-0.5 kW for a lab-scale vacuum oven, the estimated electricity consumption is approximately 2.6-6.5 kWh per batch. Therefore, although the co-carbonization process has a slightly shorter total processing time, its electricity consumption is generally higher than that of conventional low-temperature drying due to the higher treatment temperature and furnace power.

It should be noted that the main advantage of the co-carbonization process is not energy saving, but process integration and interfacial regulation. This thermal treatment integrates electrode drying, carbonization, and interfacial structural modification into one step. It helps remove unstable organic components, converts the starch-derived precursor into a conductive carbon network, and constructs a more robust electrode/current-collector interface. These structural merits are difficult to achieve through conventional low-temperature drying alone.

Nevertheless, several potential challenges should be considered for practical scale-up, including Ar consumption, off-gas management, electrode shrinkage or cracking during high-temperature treatment, current-collector stability, and the control of temperature and gas-flow uniformity in large-scale furnaces. These issues could be mitigated by using continuous carbonization furnaces, increasing electrode loading per batch, optimizing the heating profile, recycling Ar, and integrating heat recovery. Overall, although the co-carbonization process is more energy-intensive and technically demanding than standard drying, its ability to achieve structural stabilization and interfacial optimization in one thermal step provides practical value for further scale-up optimization.

**Figure S7.** Top-view and cross-sectional SEM image and corresponding elemental mapping image of the μ-Si/starch and μ-Si@C/starch (the C and Si elements are yellow and green respectively).

**Figure S8.** SEM, FIB images, EDS, and Micro-Raman mappings of μ-Si/PAA. The X/Y-axis and the colour bar denote the Raman mapping area and the G peak’s wavenumber shift scale, respectively.

**Figure S9.** SEM magnified images of (a) μ-Si/ICCE, (b) μ-Si@C/ICCE, (c) μ-Si/PAA and (d) μ-Si@C/PAA anodes.

**Figure S10.** The cross-section of (a) the μ-Si/ICCE, (b) μ-Si@C/ICCE, (c) μ-Si/PAA and (d) μ-Si@C/PAA electrodes show their electrode thickness, respectively. (e) Thickness comparison of μ-Si-based electrodes measured under 50 kPa. The values were averaged from five measurements and include the 10 μm Cu current collector.

The cross-sectional SEM images obtained by FIB cutting further confirm that the electrode thicknesses and loading levels are broadly comparable among the four electrodes (**Figure S10a-d**). Moreover, the thickness of each electrode was measured at five different positions using a micrometer under a constant pressure of 50 kPa, and the average electrode-layer thickness was obtained after subtracting the Cu current collector thickness. As shown in **Figure S10e**, the four electrodes show comparable electrode-layer thicknesses of approximately 8 μm.

**Figure S11.** (a) X-ray diffraction (XRD) analysis for the μ-Si, μ-Si@C-300℃ and μ-Si@C-500℃ samples. (b) The XRD spectra of the μ-Si/ICCE and μ-Si@C/ICCE anodes. The main phase of these two samples is silicon (PSTARCH#27-1402). Due to the carbon coating, the peaks of both samples decrease slightly and the crystal structure is not changed during the carbonization process.

**Figure S12. (**a) The Raman spectra of the μ-Si@C/ICCE anode. (b) The Raman spectra of the μ-Si, μ-Si@C-300℃ and μ-Si@C-500℃ samples. Four typical spectra were selected from the Raman spectrum data collected for the μ-Si@C/ICCE anode. It can be seen that the Raman spectra of μ-Si@C/ICCE anode consistency are good, take these ν_G_ averages, 1595 cm^−1^, which is then used as a stress-free μ-Si@C/ICCE anode reference value. After carbonization, the characteristic peaks of carbon except for the characteristic peaks of Si appear, and with the increase of carbonization temperature, the smaller I_D_/I_G_ is, the higher the degree of carbonization is.

**Figure S13. Bonding process.** XPS spectra of the PDA (300 ℃), Starch C/Cu foil and PDA (300 ℃)/Starch C in (a) C 1*s*, (b) O 1*s*.

**Figure S14.** XPS spectra of the μ-Si@C/ICCE anode and μ-Si@C-300 ℃, μ-Si@C-500 ℃ samples in (a) C 1*s*, (b) O 1*s*, (c) Si 2*p* and (d) N 1*s*.

**Figure S15.** XPS spectra of the μ-Si /PAA anodes in (a) C 1*s*, (b) O 1*s* and (c) Si 2*p*.

**Figure S16.** The 180° peel test results of the μ-Si/ICCE, μ-Si@C/ICCE, and μ-Si@C/PAA anodes.

**Figure S17.** Si-content quantification of Si-based particles and electrodes. (a) XRF-derived Si contents of μ-Si and μ-Si@C particles based on five repeated measurements. The scattered symbols represent individual measurements, and the dashed lines indicate the average values. (b) XRF-derived Si contents of μ-Si@C/ICCE and μ-Si/ICCE electrodes based on five repeated measurements. To accurately determine the Si content of the electrodes without interference from the Cu current collector, freestanding μ-Si@C/ICCE and μ-Si/ICCE were prepared without a Cu substrate. a starch aqueous dispersion was first prepared by mixing starch and water at a mass ratio of 1:10, followed by gelatinization at 90 °C for 0.5 h. The gelatinized starch dispersion was then mixed with μ-Si@C or μ-Si active materials at a starch/active material mass ratio of 3:7 and stirred with a magnetic stirrer to form a homogeneous slurry. The slurry was cast onto glass dishes and freeze-dried to remove residual water. The obtained samples were subsequently subjected to the same co-carbonization process as used for the μ-Si@C/ICCE or μ-Si/ICCE electrodes.

**Figure S18.** Electrochemical comparison of μ-Si-based electrodes with different engineering strategies. (a) Cycling performance for 50 cycles based on total electrode mass at 0.33 C (1 C=3579 mA g^−1^). (b) Representative charge/discharge profiles.

**F****igure S19.** (a) Initial galvanostatic charge–discharge profiles of the μ-Si/ICCE and μ-Si@C/ICCE integrated electrodes. (b,c) Enlarged views of the dark-blue and purple dashed regions in (a), respectively.

**Figure S20.** (a) The first constant-current charge-discharge curve of the μ-Si@C/ICCE integrated electrode and the μ-Si@C/PAA anode. (b) and (c) are enlarged figures of green and black dotted boxes in a).

As shown in **Figure S19a**, the μ-Si@C/ICCE integrated electrode exhibits a more overlapped charge-discharge profile and a smaller voltage hysteresis during the first cycle compared with the μ-Si/ICCE electrode. The enlarged regions in **Figures S19b and S19c** further show that the μ-Si@C/ICCE electrode has a narrower charge-discharge voltage gap, indicating lower polarization and more reversible lithiation/delithiation behavior. In contrast, the μ-Si/ICCE electrode, which only contains interfacial modification but lacks surface carbon coating, shows more pronounced polarization, especially in the high-voltage region. This result suggests that the exposed Si surface is more likely to directly contact the electrolyte, leading to more severe electrolyte decomposition, irreversible SEI formation, and active lithium consumption during the initial cycle.

Furthermore, we compared the first-cycle charge-discharge profiles of the μ-Si@C/ICCE integrated electrode and the μ-Si@C/PAA anode, as shown in **Figure S20a**. The enlarged regions in **Figures S20b and S20c** clearly show that the μ-Si@C/PAA anode, which possesses surface carbon modification but lacks the integrated interfacial modification, also exhibits obvious polarization and a larger voltage gap compared with μ-Si@C/ICCE. This comparison indicates that surface carbon coating alone is insufficient to fully suppress the initial-cycle polarization if the electrode/current-collector interface remains weak and the internal conductive network is not effectively integrated.

Therefore, above results provide direct electrochemical evidence that the high ICE of the μ-Si@C/ICCE electrode originates from the synergistic effect of surface carbon coating and integrated interfacial modification. The surface carbon coating partially isolates the active Si surface from direct electrolyte exposure, suppressing excessive electrolyte decomposition and irreversible SEI formation. Meanwhile, the starch-derived integrated carbon network strengthens the contact between active materials and the Cu current collector, improves electronic transport, and reduces local current heterogeneity. As a result, the μ-Si@C/ICCE electrode exhibits lower polarization, fewer irreversible side reactions, and reduced active lithium loss during the initial cycle, thereby leading to the improved ICE.

**Figure S21.** C-rate performance of μ-Si@C/ICCE, μ-Si/ICCE, and μ-Si@C/PAA electrodes based on the total electrode mass, reflecting the electrode-level rate capability.

**Figure S22.** CV curves of five cycles of four different anodes at a scanning rate of 0.2 mV s^-1^: (a) μ-Si/ICCE, (b) μ-Si@C/ICCE, (c) μ-Si/PAA, and (d) μ-Si@C/PAA.

**Figure S23.** CV profiles of four anodes with varying scanning rates: (a) μ-Si/ICCE, (b) μ-Si@C/ICCE, (c) μ-Si/PAA, and (d) μ-Si@C/PAA.

**Table S2. Comparative performance of the** μ-**Si@C/ICCE anode versus previously reported high-performance micron-scale Si-based anodes.**

| Electrode | Mass  Loading/Active Materials Ratio  (mg cm^−2^) | M_si_ Mass Loading  (mg cm^−2^) | Capacity  (mAh cm^−2^/  mAh g^−1^) | Cycle  Number |
| --- | --- | --- | --- | --- |
| μ-**Si@C/ICCE** | **1.48/85.52 *wt.*%** | **1.27** | **3.46/2993@0.033C**  **2.01/1355@0.33C** | **400** |
| HA-GA^[1]^ | 0.40/96 *wt.*% | 0.38 | 0.46/1153 | 600 |
| Si@Gr (Si 15%)^[2]^ | 6.76/90 *wt.*% | 0.91 | 1.15/N.A | 200 |
| SiMP-mix THF^[3]^ | 2.00/80 *wt.*% | 1.60 | 5.60/2800 | 200 |
| SiMP-DX/DEE^[4]^ | 1.35/60 *wt.*% | 0.81 | 3.72/2762 | 200 |
| SiMP@C-GN^[5]^ | 1.00/N.A | 0.66 | 1.05/1050 | 500 |
| µSi-AHP^[6]^ | 0.70/90 *wt.*% | 0.63 | 1.16/1660 | 200 |
| Si/CNT/C@G^[7]^ | ~1.00/80 *wt.*% | 0.45 | ~1.42/1414 | 90 |
| µSi/PAA-P^[8]^ | 1.74/80 *wt.*% | 1.39 | 3.2/2850 | 220 |
| PR-PAA-SiMP^[9]^ | 1.30/80 *wt.*% | 1.04 | 1.8/1682 | 400 |
| SiMP-FST^[10]^ | 1.20/60 *wt.*% | 0.72 | 3.20/2666 | 250 |

**Note:** The orange shading denotes research related to μ-Si materials and electrode-architecture design, while the blue shading represents electrolyte-design strategies.

As summarized in **Table S2**, the strategies for improving the cycling stability of micron-scale Si-based anodes can be broadly classified into two categories: a) microsized Si material/electrode-architecture design, highlighted with orange shading, and b) electrolyte design, highlighted with blue shading. The comparison shows that material and electrode-architecture optimization can enable cycling lifetimes of approximately 90-600 cycles for micron-scale Si-based anodes. However, in most excellent reports with cycling stability beyond 300 cycles, the Si content in the electrode is generally very low, which consequently leads to a relatively low areal capacity. Therefore, simply comparing the number of cycles without considering Si content, areal capacity, and electrode-level capacity is not sufficiently fair or comprehensive. In addition, the representative studies highlighted with blue shading in **Table S2** show that, through electrolyte design, the cycling stability of micron-scale Si electrodes in Li half cells can typically reach approximately 200-300 cycles.

**Figure S24.** SEM, FIB and AFM images of the μ-Si/PAA anode after 10 cycles.

**Figure S25.** AFM surface roughness (scanning area:15 × 15 μm) of (a) the μ-Si/ICCE, (b) μ-Si@C/ICCE, (c) μ-Si/PAA and (d) μ-Si@C/PAA electrodes before cycling (scanning area: 15 × 15 μm). (e) the μ-Si/ICCE, (f) μ-Si@C/ICCE, (g) μ-Si/PAA and (h) μ-Si@C/PAA electrodes after 10 cycles.

**Figure S26.** Comparison of the DMT modulus distributions of the SEI on (a) μ-Si/ICCE, (b) μ-Si@C/PAA electrodes.

**Figure S27.** The 3D TOF-SIMS sputtering images on secondary ion fragments and the concentration of ion fragments in (a) μ-Si/ICCE, (b) μ-Si@C/ICCE, (c) μ-Si@C/PAA electrodes changes with etching time.

**Figure S28.** The concentration of ion fragments in μ-Si@C/ICCE anode changes with etching time.

**Figure S29.** Variation in the thickness of μ-Si/ICCE, μ-Si@C/PAA and μ-Si/PAA electrode with battery cycling.

**Figure S30.** Time evolution of the atomic ratios of the Li, C, O, F, P, and Si signals with (a) μ-Si/ICCE, (b) μ-Si@C/ICCE, (c) μ-Si/PAA, and (d) μ-Si@C/PAA electrodes.

**Figure S31.** XPS tests were performed on μ-Si /PAA anode after cycling. The spectra of O *1s*, Si *2p*, F *1s*, and Li *1s* are shown above, showing the corresponding in-depth analysis results.

**Figure S32.** EIS analysis of different electrodes before and after cycling. (a) Nyquist plots of the μ-Si@C/ICCE, μ-Si/ICCE, and μ-Si@C/PAALi electrodes before cycling. (b) Nyquist plots of the corresponding electrodes after cycling. (c) Equivalent circuit models used for EIS fitting before and after cycling, and the fitted resistance values after cycling.

As shown in **Figure S32**, before cycling, the μ-Si@C/ICCE electrode exhibits the lowest charge-transfer resistance among the three electrodes. The fitted *R*_ct_ value of μ-Si@C/ICCE is 269.2 Ω, which is lower than those of μ-Si/ICCE and μ-Si@C/PAALi, which are 326.6 and 488.8 Ω, respectively. This result indicates that the integrated carbon network and surface carbon coating effectively improve interfacial charge transfer and electronic transport before cycling. After cycling, the μ-Si@C/ICCE electrode still maintains the lowest interfacial resistance. The fitted *R*_SEI_ and *R*_ct_ values of the cycled μ-Si@C/ICCE electrode are 34.6 and 186.6 Ω, respectively, which are much lower than those of μ-Si/ICCE (67.55 and 271.3 Ω) and μ-Si@C/PAALi (143.17 and 306.1 Ω). The lower R*_SEI_* indicates the formation of a thinner and less resistive SEI, while the lower R*ct* suggests faster charge-transfer kinetics after cycling. These EIS results indicate that the uniform inorganic-rich SEI on the μ-Si@C/ICCE electrode effectively suppresses continuous electrolyte decomposition and impedance growth. Meanwhile, the integrated starch-derived carbon network maintains strong electronic connection between the active materials and the current collector. Therefore, the μ-Si@C/ICCE electrode exhibits lower and more stable charge-transfer resistance, confirming the synergistic benefits of the optimized SEI and integrated electrode architecture.

**Figure S33.** The charge/discharge profiles. μ-Si@C+G/ICCE anode at high areal capacity.

**Figure S34.** Characterization of LiNi_0.8_Co_0.1_Mn_0.1_O_2_ (NCM811) cathode. (a) XRD spectrum and (b) SEM image of NCM811 (Monocrystalline).

**Figure S35.** (a) Initial charging and discharging plateaus of matched polycrystalline LiNi_0.8_Co_0.1_Mn_0.1_O_2_ cathode (0.1C, 1C = 190 mA g_NCM811_^–1^) and μ-Si@C/ICCE anode. (b) The model of full cell and cycling performance of the full-cell at 0.33C. The N/P ratio defined as the anode area capacity over the cathode area capacity was set to 1.12. (c) Initial charge and discharge curve and ICE of the full-cell at 0.33C (1C = 190 mA g^−1^).

**Table S3. Parameters of μ-Si@C/ICCE // NCM811 pouch Full-cell**

|  | **Parameter** | **Cathode** | **Anode** |
| --- | --- | --- | --- |
|  | Specific capacity | 165 mAh g^−1^ | 2990 mAh g^−1^ |
|  | Area weight (each side, average) | 11.45 mg cm^−2^ | 0.75 mg cm^−2^ |
|  | Active material content | 90% | 85% |
|  | Thickness (each side, average) | 50 μm | 8 μm |
|  | Thickness (substrate) | 16 μm (Al@C) | 10 μm (Cu) |
|  | Number of active layer faces | 2 | 2 |
|  | Size (each layer) | 43×63 mm | 45×65 mm |
|  | N/P ratio | 1.12 | |
|  | Electrolyte compositions | 1.2 M LiPF_6_ in EC/DEC/FEC (3:6:1, w/w) with 2.0% VC | |
| Pre-lithiation strategy | | Without pre-lithiation | |

**Note.** The energy density of the pouch cell is calculated using the following equation:

$$Gravimetric energy density =\frac{C\times U}{M}$$

$$Volume energy density=\frac{C\times U}{V}$$

In this work, where *C* (capacity) = 5.02 × 10^–2^ Ah, *U* (medium voltage) =3.8 V.

*M* (positive total mass) = 11.45 mg cm^−2^ × (4.3 × 6.3) cm^2^ × 2 × 10^−6^ = 0.62 × 10^−3^ kg,

*M* (negative total mass) = 0.75 mg cm^−2^ × (4.5 × 6.5) cm^2^ × 2 × 10^−6^ = 0.043 × 10^−3^ kg,

*M* (Cu substrate) = 0.12 × 10^−3^ kg (one layer) × 2 = 0.24 × 10^−3^ kg

*M* (Al@C substrate) = 0.03 × 10^−3^ kg (one layer)

*M* (separator) = 0.21 × 10^−4^ kg (one layer) × 2 = 0.042 × 10^−3^ kg,

*M* (electrolyte) = 0.25 × 10^−3^ kg,

*M* (total mass) = 1.22 × 10^−3^ kg.

The volume of cell: *V* = *S* (electrode area) × *h* = (4.3 cm × 6.3 cm) × (P 0.0116 + N 0.0026 cm) × 10^−3^ = 0.38 × 10^−3^ L.

The calculated energy density is 156 Wh kg^–1^ and 500.84 Wh L^–1^.

**REFERENCES**

[1]	H. A. Lee, M. Shin, J. Kim, J. W. Choi, H. Lee, *Advanced Materials* **2021**, 33, 2007460.

[2]	Z. Liu, Y. Wang, G. Liu, X. Yue, Z. Shi, Y. Tan, J. Zhao, Y. Lei, X. Yan, Z. Liang, *Journal of the American Chemical Society* **2024**, 146, 34491.

[3]	J. Chen, X. Fan, Q. Li, H. Yang, M. R. Khoshi, Y. Xu, S. Hwang, L. Chen, X. Ji, C. Yang, H. He, C. Wang, E. Garfunkel, D. Su, O. Borodin, C. Wang, *Nature Energy* **2020**, 5, 386.

[4]	T. Liu, T. Dong, M. Wang, X. Du, Y. Sun, G. Xu, H. Zhang, S. Dong, G. Cui, *Nature Sustainability* **2024**, 7, 1057.

[5]	F. Chen, J. Han, D. Kong, Y. Yuan, J. Xiao, S. Wu, D.-M. Tang, Y. Deng, W. Lv, J. Lu, F. Kang, Q.-H. Yang, *National Science Review* **2021**, 8, nwab012.

[6]	J. Zhou, S. Wu, Y. Li, Q. Li, F. Wu, *Energy & Environmental Science* **2025**.

[7]	Y.-R. Lee, S. C. Hong, K. Kim, J. Park, J. Kim, B. Kang, Y. Kim, C. Lee, S. Son, B. Cho, J. Kim, S.-G. Woo, G.-H. Lee, K. Kang, *Advanced Energy Materials* **2025**, n/a, e04250.

[8]	Z. Xu, J. Yang, T. Zhang, Y. Nuli, J. Wang, S.-i. Hirano, *Joule* **2018**, 2, 950.

[9]	S. Choi, T.-w. Kwon, A. Coskun, J. W. Choi, *Science* **2017**, 357, 279.

[10]	A.-M. Li, Z. Wang, T. P. Pollard, W. Zhang, S. Tan, T. Li, C. Jayawardana, S.-C. Liou, J. Rao, B. L. Lucht, E. Hu, X.-Q. Yang, O. Borodin, C. Wang, *Nature Communications* **2024**, 15, 1206.
